# Effects of Temperature Stresses on the Resistance of Chickpea Genotypes and Aggressiveness of *Didymella rabiei* Isolates

**DOI:** 10.3389/fpls.2017.01607

**Published:** 2017-09-20

**Authors:** Seid Ahmed Kemal, Sanae Krimi Bencheqroun, Aladdin Hamwieh, Muhammad Imtiaz

**Affiliations:** ^1^Biodiversity and Integrated Gene Management Program, International Center for Agricultural Research in the Dry Areas Rabat, Morocco; ^2^Centre Régional de la Recherche Agronomique de Settat, Institut National de la Recherche Agronomique Settat, Morocco; ^3^Biodiversity and Integrated Gene Management Program, International Center for Agricultural Research in the Dry Areas Giza, Egypt; ^4^International Maize and Wheat Improvement Center Islamabad, Pakistan

**Keywords:** aggressiveness, chickpea, cold, *Didymella rabiei*, pre-disposition, resistance

## Abstract

Chickpea (*Cicer arietinum* L.) is an important food and rotation crop in many parts of the world. Cold (freezing and chilling temperatures) and Ascochyta blight (*Didymella rabiei*) are the major constraints in chickpea production. The effects of temperature stresses on chickpea susceptibility and pathogen aggressiveness are not well documented in the Cicer-Didymella pathosystem. Two experiments were conducted under controlled conditions using chickpea genotypes and pathogen isolates in 2011 and 2012. In Experiment 1, four isolates of *D. rabiei* (AR-01, AR-02, AR-03 and AR-04), six chickpea genotypes (Ghab-1, Ghab-2, Ghab-3, Ghab-4, Ghab-5 and ICC-12004) and four temperature regimes (10, 15, 20, and 25°C) were studied using 10 day-old seedlings. In Experiment 2, three chickpea genotypes (Ghab-1, Ghab-2, and ICC-12004) were exposed to 5 and 10 days of chilling temperature exposure at 5°C and non-exposed seedlings were used as controls. Seedlings of the three chickpea genotypes were inoculated with the four pathogen isolates used in Experiment 1. Three disease parameters (incubation period, latent period and disease severity) were measured to evaluate treatment effects. In Experiment 1, highly significant interactions between genotypes and isolates; genotypes and temperature; and isolate and temperature were observed for incubation and latent periods. Genotype x isolate and temperature x isolate interactions also significantly affected disease severity. The resistant genotype ICC-12004 showed long incubation and latent periods and low disease severity at all temperatures. The highly aggressive isolate AR-04 caused symptoms, produced pycnidia in short duration as well as high disease severity across temperature regimes, which indicated it is adapted to a wide range of temperatures. Short incubation and latent periods and high disease severity were observed on genotypes exposed to chilling temperature. Our findings showed that the significant interactions of genotypes and isolates with temperature did not cause changes in the rank orders of the resistance of chickpea genotypes and aggressiveness of pathogen isolates. Moreover, chilling temperature predisposed chickpea genotypes to *D. rabiei* infection; developing multiple stress resistance is thus a pre-requisite for the expansion of winter-sown chickpea in West Asia and North Africa.

## Introduction

Chickpea (*Cicer arietinum* L.) is an important cool-season food legume crop grown in many parts of the world. The crop is produced for local consumption, to generate export earnings and act as a break crop to improve soil fertility and health. Chickpea is mainly grown in spring in many parts of the world where drought, heat, wilt/root rots, Ascochyta blight and insect pests limit crop productivity and production (Singh et al., 1989; Jha et al., 2014; Li et al., 2015). Chickpea yield can be substantially increased by adopting early winter sowing at low to medium altitudes in the West Asia and North African (WANA) region (Hawtin and Singh, 1984; Singh et al., 1989; Mazid et al., 2013). However, sowing chickpea in winter can increase the risk of exposing the crop to subzero temperatures as low as −10°C for up to 60 days and to chilling temperature and Ascochyta blight epidemics during the cropping season (Malhotra and Singh, 1991; Singh et al., 1993; Croser et al., 2003; Nezami et al., 2012).

To tap the potential of winter chickpea sowing in low to medium altitude areas, the International Center for Agricultural Research in the Dry Areas (ICARDA) has initiated a chickpea breeding program targeting winter sowing since the 1974/75 cropping season. The two key traits for the success of winter sown chickpea are cold (freezing) tolerance at the seedling and vegetative stages and resistance to Ascochyta blight (*Didymella rabiei*). ICARDA develops cold tolerant chickpea germplasm in key cold testing sites in Syria (Tel Hadya Research Station), Turkey (Eskishehr Research Station), Lebanon (Terbol Research Station) and Iran (Maraghahe Research Station). Breeding lines and germplasm accessions (cultivated and wild relatives) are exposed to freezing temperatures ranging from −5°C in Lebanon and Syria to −20°C in Turkey and Iran. The first cultivars released for winter sowing with cold tolerance and Ascochyta blight resistance were ILC-482 and ILC-3279 (Malhotra and Singh, 1991; Singh et al., 1992a,b). The cultivar ILC-482 has moderate levels of Ascochyta blight resistance and tolerance to freezing temperatures as low as −10°C and can yield up to 4 t/ha (Hawtin and Singh, 1984). Besides high productivity, winter-sown chickpea cultivars are taller than spring sown chickpea and allows mechanical harvesting that solves labor shortages during harvesting and threshing (Singh et al., 1997). Moreover, wilt/root rots and leaf miner are less problematic in winter-sown than in spring-sown chickpea crops.

Ascochyta blight is a major biotic factor contributing to high yield gaps in chickpea in many countries (Pande et al., 2005; Singh et al., 2007). The pathogen is heterothallic and requires the pairing of two compatible mating types (MAT1-1 and MAT1-2) for sexual reproduction. In the presence of the two mating types, fertile pseudothecia can develop on overwintering chickpea straw; ascospores act as primary inoculum sources to initiate disease foci that can lead to epidemics under favorable environmental conditions. Many races and pathotypes of *D*. *rabiei* have been reported in the WANA region (Udupa et al., 1998; Nourollahi et al., 2011; Atik et al., 2013). Different approaches are available to manage Ascochyta blight in chickpea crops with varying levels of effectiveness. These include foliar fungicide applications, seed treatment, agronomic practices, growing resistant cultivars and integration of two or more control options (Gan et al., 2006; Chang et al., 2007a; Dusunceli et al., 2007; Lobna et al., 2010). Although different Ascochyta blight management options are available for growers, breeding for host plant resistance is given the highest priority by national and international chickpea breeding programs (Singh and Reddy, 1996; Muehlbauer and Chen, 2007; Rubiales and Fondevilla, 2012; Sharma and Ghosh, 2016). The resistance of cultivars released in many countries is controlled by both major and minor genes (Lichtenzveig et al., 2002; Muehlbauer and Chen, 2007; Rubiales and Fondevilla, 2012; Labdi et al., 2013; Sharma and Ghosh, 2016). For example, the first two cultivars (genotypes ILC-482 and ILC-3279) released for winter sowing in many countries in WANA region have rate reducing resistance to the *D. rabiei* population existing in the early 1980s in Syria (Reddy and Singh, 1993) but this resistance was eroded through the appearance of more aggressive pathogen populations (Imtiaz et al., 2011; Atik et al., 2013; Hamwieh et al., 2013).

Interactions between biotic and abiotic stresses have been reported in many host-pathogen pathosystems where plants exposed to abiotic stresses showed either increased or decreased resistance/tolerance to subsequent infections by pathogens (Atkinson and Urwin, 2012; Bostock et al., 2014; Suzuki et al., 2014; Moyer et al., 2015). Temperature has a significant effect on host plant resistance genes and pathogen virulence and aggressiveness in many pathosystems. For example, in lupin, resistant cv. Wonga became susceptible to anthracnose when the temperature increased from 12–18° to 26°C (Thomas et al., 2008). When infected with different *Fusarium oxysporum* f. sp. *ciceris* races, chickpea cv. Ayala was moderately resistant at 24/21°C but susceptible at 27/25°C (Landa et al., 2006). In wheat, some isolates of *Puccinia striiformis* f. sp. *tritici* showed increased aggressiveness at high temperature (Milus et al., 2009). In sunflower, resistance to *Orobanche aegyptiaca* was found to be temperature dependent (Eizenberg et al., 2003). Studies on biotic-abiotic interactions in *Ascochyta*-legume pathosystem have mainly focused on the role temperature and wetness period play in inoculum production and disease development (Trapero-Casas and Kaiser, 1992, 2007; Pedersen and Morrall, 1994; Roger et al., 1999; Tivoli and Banniza, 2007; Golani et al., 2016), but studies on the role temperature stress plays in host resistance, pathogen aggressiveness and predisposing chickpea to pathogen infection are lacking. Therefore, this study was designed to assess: (1) the effect of different temperature ranges on host resistance and pathogen aggressiveness in *Cicer-Didymella* pathosystem; and (2) how chilling temperature pre-disposes chickpea genotypes to *D. rabiei* infections.

## Materials and methods

Two independent experiments were conducted in 2011 and 2012 at ICARDA Tel Hadya Research Station, Syria. Experiment 1 was conducted in two controlled environment growth cabinets (Conviron Model E15, Winnipeg, Canada), adjusted to a 16-h photoperiod of approximately 450 mmol m^−2^ s^−1^ light intensity provided by fluorescent light (Jhorar et al., 1998; Udupa et al., 1998); Experiment 2 was conducted under plastic house conditions. In both experiments, four *D. rabiei* isolates, namely, AR-01 (Pathotype-1, weakly aggressive), AR-02 (Pathotype-2 moderately aggressive), AR-03 (Pathotype-3, aggressive) and AR-04 (Pathotype-4, highly aggressive), were used (Udupa et al., 1998; Imtiaz et al., 2011). The four isolates are routinely used to screen ICARDA chickpea breeding lines for Ascochyta blight resistance under field and controlled conditions (Hamwieh et al., 2013).

### Effects of temperature on host resistance and pathogen aggressiveness (Experiment 1)

Four temperature regimes (10, 15, 20, and 25°C) and five released Kabuli chickpea genotypes for winter sowing, namely Ghab-1 (ILC-482), Ghab-2 (ILC-3279), Ghab-3 (FLIP-150-82), Ghab-4 (FLIP-85-122), Ghab-5 (FLIP-88-85), and one desi chickpea genotype (ICC-12004) were used in this experiment. All released cultivars in this study were resistant to pathogen populations existing during the year of their release. Seedlings (five seeds per 10-cm diameter pot) of each genotype planted in a sterilized soil-peat moss mixture in plastic house at 20 ± 2°C for 10 days were used for this experiment. The experiment was laid out in a split-split plot design (main plot: temperature; sub-plot: genotypes and sub-sub plots: isolates) with three replications and performed four times. The four *D. rabiei* isolates were grown on chickpea dextrose agar (4% chickpea flour; 2% dextrose and 2% agar in 1 l of distilled water) for 7–10 days inside a culture room at 21–23°C under 16/8 h light and darkness cycles. Spore suspensions were prepared the day of seedling inoculations by flooding the surface of the culture in Petri dishes with distilled water and then scraping the surface of the culture with a glass rod to release spores from pycnidia. Spore concentrations were determined using a Neubauer hemacytometer and adjusted to the desired concentration by diluting with distilled water. Ten-day old chickpea raised in the plastic house were inoculated with a spore suspension (5 × 10^5^ spores m^−*l*^) of each isolate using hand sprayers until runoff for each temperature regime. Inoculated seedlings were covered with transparent polyethylene sheets for 72 h. After removing the plastic covers, seedlings were misted 3–4 times daily with distilled water to maintain favorable conditions (leaf wetness and 70% or more relative humidity in the growth cabinets) and favor disease development.

### Effect of chilling temperature in predisposing chickpea genotypes to *D*. *rabiei* infection (Experiment 2)

**Figure 1:**
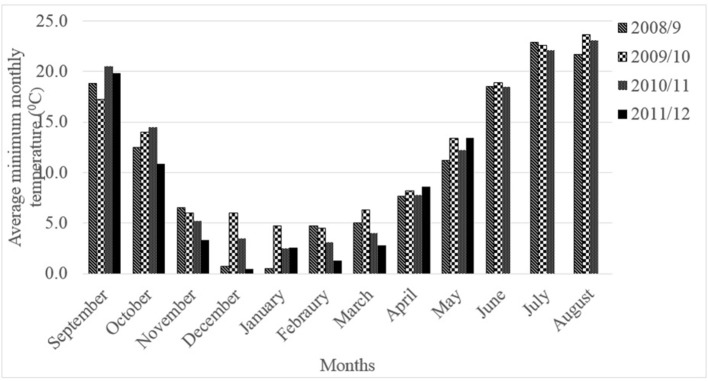
Average monthly minimum temperature (°C) at ICARDA Tel Hadya Research Station, 2008–2012 cropping seasons.

Three chickpea genotypes (Ghab-1, Ghab-3, and ICC-12004) were used in this experiment. Cultivars Ghab-1 and Ghab-3 are tolerant to cold but the reaction of genotype ICC-12004 is not documented. The isolates, number of seedlings per pot, experimental design, number of replications, spore concentrations and inoculation and incubation methods were similar to those described under **Experiment 1**. Seedlings (10 day-old) were raised in a plastic house at 20 ± 2°C and 16/8 h light/dark cycles and exposed to chilling temperature (5°C) in a cold chamber (16/8 h light/ dark and light cycles) for 5 and 10 days before inoculation. After chilling exposure, seedlings were returned to the plastic house for inoculation with pathogen isolates. Sets of seedlings of each genotype not exposed to chilling temperature and raised at 20 ± 2°C in a plastic house were used as controls. High humidity (>75%) was kept in the plastic house by misting water for 30 s at 2-3-h intervals. The chilling temperature (5°C) was selected because the cold chamber cannot be adjusted at a lower temperature; the temperature closer to the low temperatures prevailing during the seedling and vegetative stages of winter-sown chickpea at ICARDA, Tel Hadya Research Station, Syria (Figure 1) and based on published works on cold tolerance research on chickpea (Nayyara et al., 2005; Bakht et al., 2006). The Tel Hadya Research Station experiences 17–56 days of freezing temperature (Malhotra and Singh, 1991; Singh et al., 1993).

### Disease parameters and data analyses

Three disease parameters, incubation period (IP) as the interval between inoculation and the first appearance of symptoms; latent period (LP) as the interval between inoculation and the first appearance of pycnidia on infected seedlings; and disease severity were recorded for seedlings in each pot in both experiments. Disease severity was recorded using a 1–9 rating scale where 1 = healthy plant, no disease; 2 = lesions present, but small and inconspicuous; 3 = lesions easily seen, but plants are mostly green; 4 = severe lesions clearly visible, stem infection is clear; 5 = lesions girdle stems, most leaves show lesions; 6 = plants collapsing, tips die back; 7 = plants dying, but at least three green leaves present; 8 = nearly all plants dead but still have a green stem; and 9 = dead plants (Chen et al., 2004). Final disease severity (the average of the scores in 5 plants per pot) was scored approximately 20 days after inoculation in Experiment-1 and 15 days after inoculation in Experiment-2 after one disease cycle was completed. All data for the three disease parameters were analyzed using the residual (restricted) maximum likelihood (REML) method in a generalized linear mixed model (Garrett et al., 2004; Onofri et al., 2010) using Genstat Software (16th edition). In the model, temperature, duration of chilling temperature exposure, genotypes and isolates were assigned as fixed effects and number of times the experiments were repeated and replications as random effects. Least square differences were calculated from the standard errors of the differences for mean comparisons. Correlation analyses were made between disease parameters.

## Results

### Effects of temperature on host resistance and pathogen aggressiveness

The variance component analyses for fixed effects of the three disease parameters are presented in Table 1. Interactions between genotypes and isolates; genotypes and temperature, and isolates and temperature were highly significant for IP and LP. Highly significant interactions were observed for genotype and isolate and isolate and temperature interactions for disease severity. No significant differences were observed between genotypes and temperature for disease severity.

**Table 1:** Generalized linear mixed model analysis of fixed factors for three disease parameters on chickpea genotypes inoculated with *Didymella rabiei* isolates and incubated at different temperature ranges.

**Fixed factors**	**Degree of freedom**	**Incubation period**	**Latent period**	**Disease severity**
Genotypes (G)	5	*P* < 0.001	*P* < 0.001	*P* < 0.001
Isolates (I)	3	*P* < 0.001	*P* < 0.001	*P* < 0.001
Temperature (T)	3	*P* < 0.001	*P* < 0.001	*P* < 0.001
GXI	15	*P* < 0.01	*P* < 0.005	*P* < 0.003
GXT	15	*P* < 0.001	*P* < 0.001	*P* < 0.061
IXT	9	*P* < 0.001	*P* < 0.001	*P* < 0.001
GXPXT	45	*P* < 0.006	*P* < 0.001	*P* < 0.003

#### Incubation and latent periods

**Figure 2:**
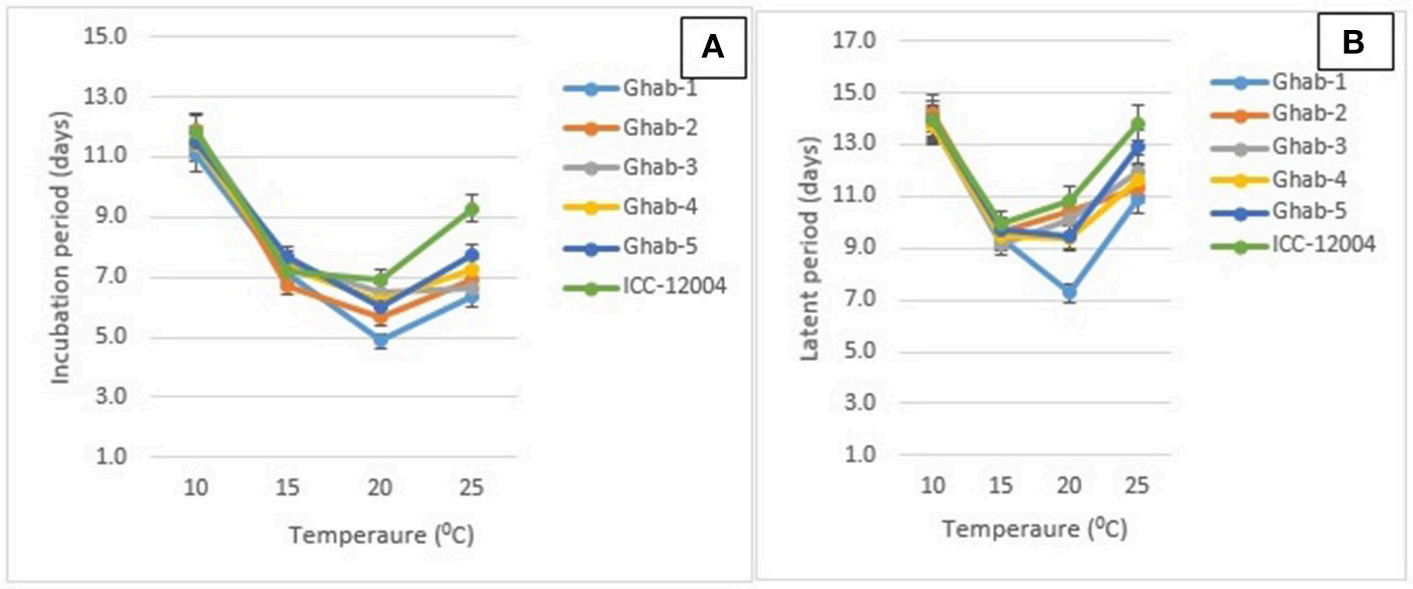
Effects of temperature on mean incubation **(A)** and latent periods **(B)** of chickpea genotypes inoculated with four isolates of *Didymella rabiei*. Vertical bars indicate standard errors of the means.

The IP among chickpea genotypes ranged from 7 to 9 days and LP ranged from 10 to 12 days (Table 2). Across all chickpea genotypes, IP and LP were the shortest when infected by isolate AR-04, and the longest when infected by AR-01. In the genotype × temperature interactions (*P* < 0.001), IP and LP were significantly longer at 10°C than the other temperatures across all chickpea genotypes (Figures 2A,B). Both IP and LP showed a decreasing trend from 15 to 20°C and increased when the temperature increased to 25°C. The shortest IP was observed in Ghab-1 and longest in ICC-12004 at 20°C. A similar trend was observed for LP where genotypes showed long LP at 10°C and then decreased during incubation at 15 and 20°C. The shortest LP was observed at 20°C on Ghab-1.

**Table 2:** Mean incubation and latent periods (days) of chickpea genotypes inoculated with four isolates of *Didymella rabiei*.

**Parameters**	**Genotypes**	**Isolates**				
		**AR-01**	**AR-02**	**AR-03**	**AR-04**	**Mean**
Incubation period	Ghab-1	8.4	7.8	7.3	6.0	7.4
	Ghab-2	8.7	7.6	7.6	7.2	7.8
	Ghab-3	9.6	8.3	8.0	6.5	8.1
	Ghab-4	9.2	9.1	7.5	6.4	8.1
	Ghab-5	8.9	9.2	8.0	6.6	8.2
	ICC-12004	9.1	9.7	8.9	7.5	8.8
	Mean	9.0	8.6	7.9	6.7	8.0
LSD (0.01) for genotype by isolate interaction = 1.05						
	Ghab-1	11.2	10.5	10.3	9.4	10.3
Latent period	Ghab-2	13.1	11.6	11.0	10.0	11.4
	Ghab-3	12.6	11.5	11.5	9.5	11.3
	Ghab-4	12.6	11.5	10.6	9.6	11.1
	Ghab-5	12.9	11.9	11.7	9.5	11.5
	ICC-12004	12.3	13.0	13.1	10.4	12.2
	Mean	12.4	11.6	11.3	9.7	11.3
LSD (0.01) for genotype by isolate interaction = 0.62						

**Figure 3:**
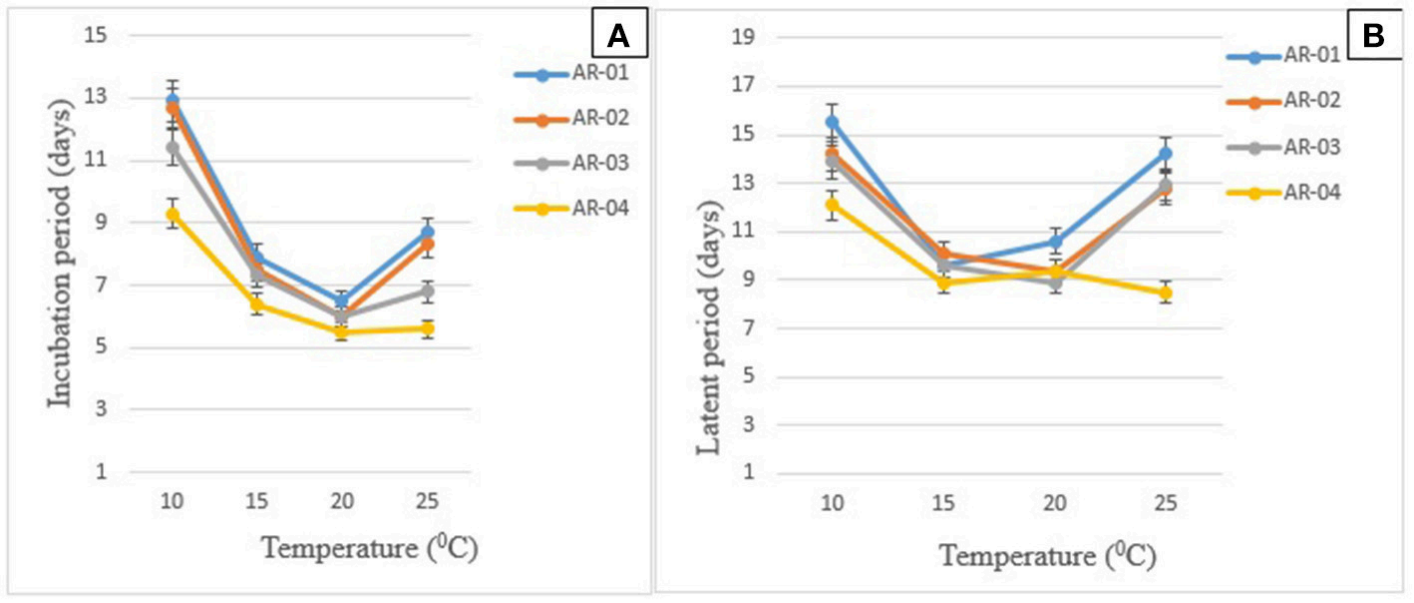
Effects of temperature on the mean duration of the development of symptoms **(A)** and pycnidia **(B)** on chickpea genotypes inoculated with four isolates of *Didymella rabiei*. Vertical bars indicate standard errors of the means.

In the isolate × temperature interaction (*P* < 0.001), all isolates took a long time to cause symptoms and produce pycnidia on chickpea genotypes incubated at 10°C (Figures 3A,B). Short IP was observed at 15–25°C for all isolates. However, except AR-04, the other isolates showed long IP at 25°C incubation temperature. Isolate AR-04 caused symptoms and produced pycnidia within a short period of time under all temperature regimes.

#### Disease severity

Overall there were differences among isolates in their aggressiveness that caused disease severity ranging from 3 to 7, while the susceptibility of genotypes was narrower, and across all isolates ranged from 5 to 6. In genotype × isolate interactions, isolates AR-04 and AR-03 caused high disease severity across all chickpea genotypes (Table 3). Isolate AR-01 showed low levels of disease severity (<4) on all genotypes. Isolate AR-03 caused high disease severity on Ghab-1 compared to its aggressiveness on the other genotypes. In isolate by temperature interactions, isolate AR-04 caused high disease severity at 10–15°C and the lowest severity at 25°C (Table 4). Isolate AR-01 caused high disease severity at 10–15°C than at the other two temperature regimes. The highly aggressive isolate AR-04 caused high disease severity at 25°C compared to the other isolates.

**Table 3:** Mean disease severity on chickpea genotypes inoculated with four isolates of *Didymella rabiei*.

**Genotypes**	**Isolates**				
	**AR-01**	**AR-02**	**AR-03**	**AR-04**	**Mean**
Ghab-1	3.5	6.1	6.5	7.5	5.9
Ghab-2	3.0	4.9	5.8	7.1	5.2
Ghab-3	3.0	4.6	5.4	7.6	5.2
Ghab-4	2.7	4.5	5.2	7.4	4.9
Ghab-5	2.7	4.8	5.3	7.2	5.0
ICC-12004	2.9	4.7	5.0	6.6	4.8
Mean	3.0	4.9	5.5	7.3	5.2
LSD (0.01) for genotype by isolate interaction = 0.56					

**Table 4:** Combined reaction of six chickpea genotypes to four isolates of *Didymella rabiei* on mean disease severity.

**Genotypes**	**Temperature regimes**				
	**10**	**15**	**20**	**25**	**Mean**
AR-01	4.0	3.5	2.2	2.2	3.0
AR-02	5.0	6.5	5.6	2.7	4.9
AR-03	5.0	6.7	5.8	4.6	5.5
AR-04	7.9	8.0	7.2	5.9	7.3
Mean	5.5	6.2	5.2	3.9	5.2
LSD (0.01) for isolate by temperature interaction = 0.56					

### Effect of chilling temperature in predisposing chickpea genotypes to *Didymella rabiei* infection

Non-significant interactions were observed between chilling duration × genotypes for LP and disease severity. Moreover, genotype × isolate interactions were not significant for IP and LP (Table 5). Highly significant interactions were observed between chilling duration and genotypes for IP; between chilling duration and isolate for the three disease parameters; and between genotype and isolates for disease severity.

**Table 5:** Generalized linear mixed model analysis of fixed factors for three disease parameters on chickpea genotypes exposed to chilling temperature.

**Fixed factors**	**Degree of freedom**	**Incubation period (days)**	**Latent period (days)**	**Disease severity**
Duration (D)	2	*P* < 0.001	*P* < 0.001	*P* < 0.001
Genotypes (G)	2	*P* < 0.001	*P* < 0.001	*P* < 0.001
Isolates (I)	3	*P* < 0.001	*P* < 0.001	*P* < 0.001
DXG	4	*P* < 0.03	*P* < 0.551	*P* < 0.706
DXI	6	*P* < 0.001	*P* < 0.001	*P* < 0.001
GXI	6	*P* < 0.104	*P* < 0.159	*P* < 0.001
DXGXI	12	*P* < 0.002	*P* < 0.360	*P* < 0.856

#### Incubation and latent periods

**Figure 4:**
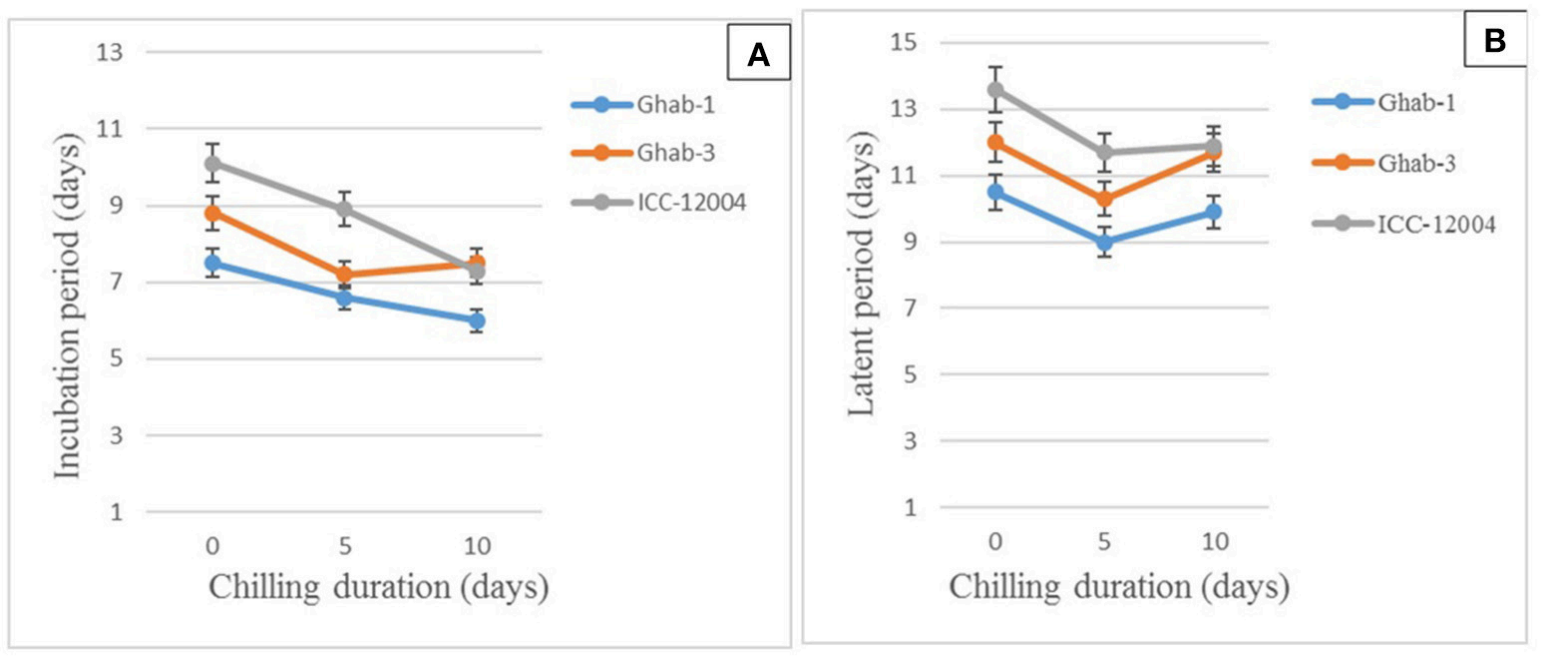
Effects of chilling duration on mean incubation **(A)** and latent periods **(B)** on chickpea genotypes inoculated with four isolates of *Didymella rabiei*. Vertical bars indicate standard errors of the means.

The mean IP for chickpea genotypes ranged from 7 to 9 days, while for LP it ranged from 10 to 12 days. Shorter IP and LP were observed on chickpea genotypes infected with the four isolates and exposed to chilling temperature than on the controls. The 5-day chilling period shortened the duration of pycnidia formation on the chickpea genotypes infected with across all isolates. In chilling duration x isolate interactions, all pathogen isolates caused symptoms within a shorter period on all chickpea genotypes exposed to chilling temperature than on the controls (Figures 4A,B). Isolates AR-01 and AR-02 caused disease symptoms within a significantly shorter period on chickpea genotypes exposed to chilling temperature than on the controls. The pycnidia formation period was shorter under chilling temperature for isolates AR-01 and AR-03, while the highly aggressive isolate AR-04 produced symptoms and pycnidia within a short period under all treatments (Table 6).

**Table 6:** Effects of chilling temperature on incubation and latent period of four isolates of *Didymella rabiei* inoculated on three chickpea genotypes.

**Parameters**	**Chilling duration (days)**	**Isolates**				
		**AR-01**	**AR-02**	**AR-03**	**AR-04**	**Mean**
Incubation period	0	13.2	8.8	7.2	6.0	8.8
	5	10.1	7.1	7.3	7.6	7.6
	10	6.9	6.7	7.6	6.6	7.0
	Mean	10.1	7.5	7.4	6.1	7.8
LSD (0.01) for chilling duration x isolate interaction = 1.44						
Latent period	0	16.7	12.3	10.7	8.5	12.1
	5	13.2	9.8	10.0	8.2	10.3
	10	9.8	10.0	12.9	9.6	10.6
	Mean	13.2	10.7	11.2	8.8	11.0
LSD (0.01) for chilling duration x isolate interaction = 1.83						

#### Disease severity

**Figure 5:**
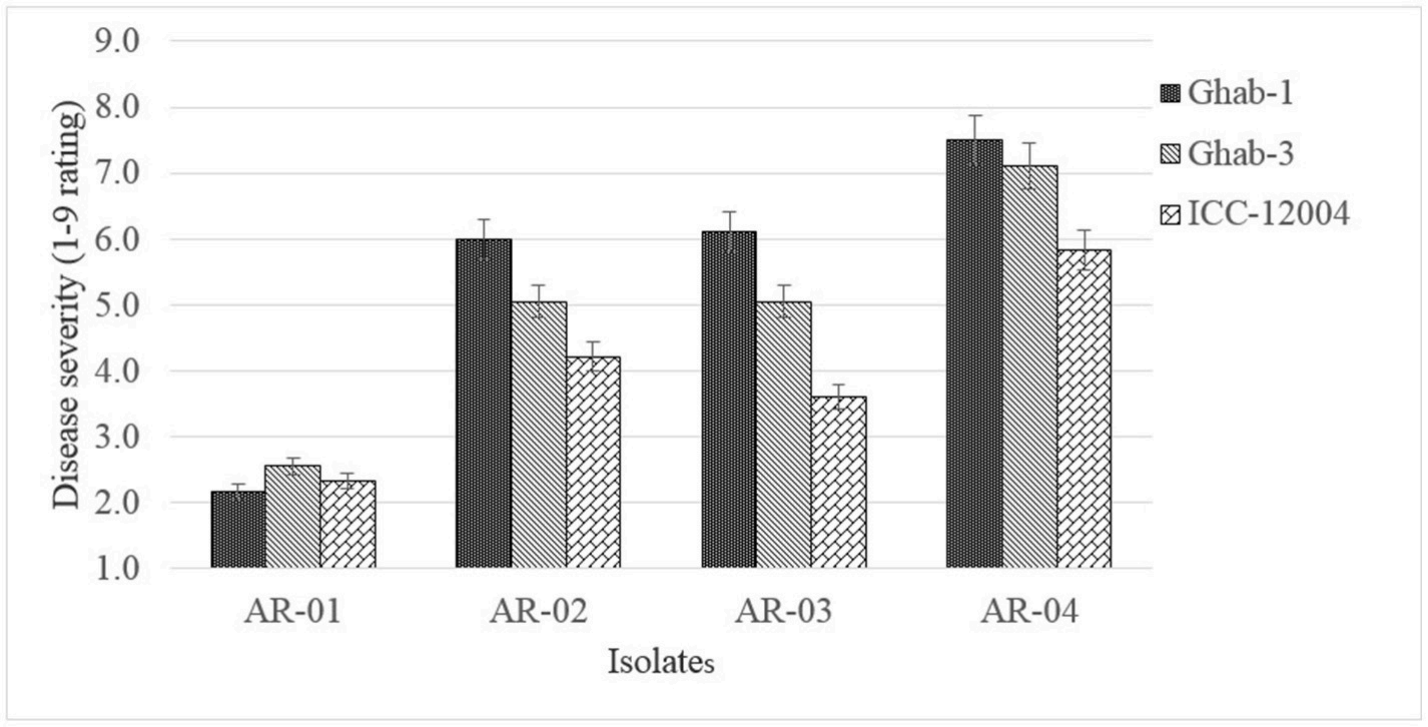
Mean disease severity in chickpea genotypes inoculated with four isolates of *Didymella rabiei*. Vertical bars are standard errors of the means.

The genotype × chilling exposure interaction did not show significant differences but genotypes Ghab-1 and ICC-12004 showed increased disease severity under chilling temperature (data not shown). In the genotype x isolate interaction (*P* < 0.003), isolate AR-04 caused high disease severity on all chickpea genotypes, while genotype ICC-12004 showed low levels of susceptibility to all pathogen isolates (Figure 5). The genotype did not show changes in rank order due to infection by *D*. *rabiei* isolates with varying levels of aggressiveness as affected by chilling temperature.

**Figure 6:**
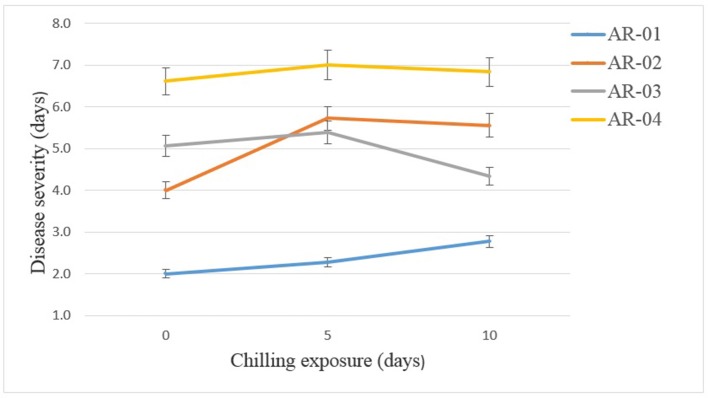
Effects of chilling exposure on the aggressiveness of *Didymella rabiei* isolates inoculated on chickpea genotypes. Vertical bars are standard errors of the means.

In the chilling duration × isolate interactions, isolate AR-04 caused high disease severity on chickpea genotypes in all treatments (Figure 6). The weakly aggressive isolate AR-01 caused more disease on chickpea exposed to chilling temperature than on the controls. Isolate AR-02 caused more disease on seedlings exposed to chilling temperature. However, isolate AR-3 caused more disease on seedling exposed to 5 days chilling temperature that 10 days exposure.

### Correlations among disease parameters

All correlations between two disease parameters were highly significant (*P* < 0.001) in both experiments. In Experiment 1, the correlation between IP and LP was positive and high (*r* = 0.71), while the correlation between LP and disease severity was intermediate and negative (*r* = −0.55). The correlation between IP and disease severity was low and negative (*r* = −0.30). In Experiment 2, the correlation between IP and LP was positive (*r* = 0.73), while the correlations between IP and disease severity (*r* = −0.63) and LP and disease severity (*r* = −0.73) were high and negative.

## Discussion

Ascochyta blight and low temperature (cold and frost) will remain key production constraints to both winter and spring sown chickpea crops in many countries in the world. Ascochyta blight epidemics depend on weather conditions (moisture and temperature), level of variety resistance and aggressiveness of the pathogen population. The effect of biotic-abiotic interactions on pre-disposing hosts and the effectiveness of resistance genes is becoming an important issue since it affects disease management practices and breeding strategies (Bostock et al., 2014). Hence, knowledge on abiotic and biotic interactions on winter-sown chickpea is important in developing appropriate disease management practices to reduce the impact of Ascochyta blight and expand winter chickpea technology in WANA.

In Experiment 1, significant genotype x isolate interactions did not show changes in resistance of chickpea genotypes to the four isolates measured based on the three disease parameters indicating that reactions of the chickpea genotypes were temperature independent. Although resistance reactions of the chickpea genotypes are based on disease severity rating, they showed differences in IP and LP that could be used as selection criteria for partial resistance in chickpea resistance breeding. Temperature also did not affect the level of aggressiveness of the isolates where the highly aggressive isolate AR-04 showed short IP, LP and high disease severity across all temperature ranges. Isolate AR-04 is highly aggressive to all available chickpea genotypes developed at ICARDA, indicating more attention in developing resistant genotypes from cultivated and wild relatives to manage highly aggressive pathogen population like isolate AR-04 in the future since many isolates with similar aggressiveness with AR-04 are reported in Syria (Atik et al., 2013). High temperature prevails during podding stage of chickpea and if supplementary irrigation is given, it provides long wetness period and isolates adapted to high temperature range can cause heavy pod and seed infections that affect seed quality and increased the chance of pathogen spread to new areas through germplasm exchanges. Under controlled conditions, Frenkel et al. (2010) found that *D. rabiei* isolates collected from cultivated chickpea showed high temperature adaptation than those isolates from wild chickpea.

In Experiment 2, chickpea genotypes showed short IP and LP and high disease severity when exposed to chilling temperature. Cold exposure can pre-dispose resistant chickpea genotypes to both weakly and highly aggressive pathogen populations and can cause disease epidemics under field conditions if conditions are favorable for disease development. The roles of abiotic stress in affecting resistance genes, increasing pathogen virulence and pre-disposing crops to subsequent pathogen attacks have been reported by many researchers (Landa et al., 2006; Thomas et al., 2008; Bostock et al., 2014). The effects of cold in pre-disposing crops to pathogen infections are reported in rice to blast (Koga et al., 2004) and cotton to Alternaria blight (Zhao et al., 2012). Our findings showed that chilling temperature can pre-dispose chickpea genotypes to *D. rabiei* infection and that could lead to blight epidemics in winter-sown crops in WANA.

The negative high and significant correlation between LP and disease severity in both experiments is an indication that isolates that produce pycnidia within a short period can cause many disease cycles compared with isolates with a long pycnidia formation period. Moreover, LP could be an important fitness component in dominating the population of *D*. *rabiei* during epidemics. This study showed no significant tradeoff between IP, LP and disease severity under controlled conditions. Incubation period was found to differentiate field pea genotypes to *Mycosphaerella pinodes* and correlated with final disease severity, area under the disease progress curve and rate of disease development under controlled condition (Prioul et al., 2003).

The chilling duration did not show a clear trend on the aggressiveness of isolates inoculated on chickpea genotypes. Some isolates showed increased aggressiveness over others and this could lead different isolates to dominate the pathogen population during the epidemic period. In wheat- *Zymoseptoria tritici* pathosystem, variations in aggressiveness were observed between initial pathogen isolates in winter and final population in spring (Suffert et al., 2015).

The expansion of winter-sown chickpea in WANA requires germplasm that tolerates freezing and chilling and has high levels of Ascochyta blight resistance. At ICARDA, separate screening germplasm and breeding lines are developed for freezing tolerance and Ascochyta blight resistance. However, Ascochyta blight screening is done by planting chickpea in December, which exposes the crop to chilling temperatures that could predispose the crop to *D. rabiei* infections when temperature increases in February-April at Tel Hadya and Terbol Research Stations. During some growing seasons, chickpea breeding lines planted for Ascochyta blight screening are hit by freezing temperature; some genotypes regenerate from cold damage but show high susceptibility to Ascochyta blight (Ahmed, personal observation). Regeneration ability could be a trait of interest for cold tolerance; thus there is a need to look for genotypes that have the ability to regenerate as well as good levels of Ascochyta blight resistance.

Pre-disposing effects of chilling temperature on partially resistant winter-sown chickpea cultivars could lead to severe Ascochyta blight epidemics that may require more fungicide spraying to manage the disease. Repeated fungicide applications will increase farmers' production costs and could create *D. rabiei* populations that are insensitive to the available fungicides. In addition to insensitivity to the fungicides most widely used in WANA, there are reports of fungicide tolerance in *D. rabiei* populations from chickpea fields in USA and Canada (Chang et al., 2007b; Wise et al., 2008). In order to mitigate climate change and variability, conservation cropping is being adopted in WANA where chickpea is a key rotation crop in cereal cropping system where infected chickpea straws are left in the farm. Hence, chilling temperature favors sexual reproduction (Navas-Corte's et al., 1998) where the two mating types of *D. rabiei* are existing. The discharge of ascospores usually coincides with the onset of the vegetative growth of the chickpea crop and infection and subsequent disease epidemics will be high on chickpea crops pre-disposed by chilling temperature during this crop stage of the crop. To improve selection for cold tolerance combined with Ascochyta blight resistance, conventional screening methods should be supported by marker-assisted selection.

This study was limited to a few temperature regimes and pathogen isolates to study the impact of temperature on host plant resistance and pathogen aggressiveness. Moreover, chilling temperature, duration of chilling exposure and crop growth stages were very limited due to shortage of space. Changing the physiological and other tolerance mechanisms to chilling temperature of the studied chickpea genotypes was not attempted. Studies showed that the natural plant growth regulators Abscisic acid (ABA) plays a role in cold tolerance in chickpea (Bakht et al., 2006; Kumar et al., 2008). However, ABA produced during chilling temperature is reported to expose crops to pathogen infections (Mauch-Mani and Mauch, 2005) but the role of ABA in pre-disposing chickpea to *D. rabiei* infection in winter planted chickpea is not known and future investigation is required.

In conclusion, our study showed that Ascochyta blight resistance in the studied chickpea genotypes and the aggressiveness of pathogen isolates are not temperature dependent. Hence, resistance screening and genetic studies can be done in temperature ranging from 15 to 20°C since resistance and aggressiveness are temperature independent. Chilling temperature pre-disposes chickpea genotypes to *D. rabiei* infections and increased disease severity. As a result, emphasis should be given to developing germplasm with high levels of cold tolerance and Ascochyta blight resistance. Our study clearly showed the existence of D. rabiei isolates that can cause high disease severity under wide ranges of temperature.

## Author contributions

SA, SK and MI planned the idea and did preliminary controlled experiments in 2011 at ICARDA Tel Hadya Research Station, Syria and later SA modified the original experiments and implemented 2011 and 2012 at the same place. Data analyses and first draft and revisions of the manuscript were done by SA, AH, MI, and SK and agreed before submissions.

### Conflict of interest statement

The authors declare that the research was conducted in the absence of any commercial or financial relationships that could be construed as a potential conflict of interest.
